# Optimization of Protein Quality of Plant-Based Foods Through Digitalized Product Development

**DOI:** 10.3389/fnut.2022.902565

**Published:** 2022-05-10

**Authors:** Zaray Rojas Conzuelo, Roger Robyr, Katrin A. Kopf-Bolanz

**Affiliations:** School of Agricultural, Forest and Food Sciences HAFL, Bern University of Applied Sciences, Bern, Switzerland

**Keywords:** plant-based proteins, optimization, protein quality, digital product development, plant-based products, linear optimization

## Abstract

With the increasing availability of plant-based protein products that should serve as alternatives to animal-based protein products, it is necessary to develop not only environmentally friendly but also nutritious foods. Especially the protein content and quality are of concern in these products. The algorithm of NutriOpt was developed using linear programming to support the development of food products with a balanced amino acid profile while considering digestibility. The current version contains a database with 84 plant protein sources from different food groups (legumes, cereals, nuts, seeds) and with different grades of purification (flours, concentrates, isolates) from which NutriOpt can create mixtures with high protein quality while complying with constraints such as protein content, number of ingredients, and weight of the mixture. The program was tested through different case studies based on commercial plant-based drinks. It was possible to obtain formulations with a Protein Digestibility Corrected Amino Acid Score (PDCAAS) over 100 with ingredients and quantities potentially suitable for plant-based analogs. Our model can help to develop the second generation of plant-based product alternatives that can really be used as an alternative on long-term consumption. Further, there is still a great potential of expansion of the program for example to use press cakes or even to model whole menus or diets in the future.

## Introduction

Environmental degradation and diet-related non-communicable diseases are only a few of the negative outcomes of the high consumption of animal-based products in Western diets (1).

Research has shown that shifting toward a more plant-based dietary pattern can potentially provide major health benefits. For instance, vegans, vegetarians, pescatarians, and semi-vegetarians had a 12 percent lower overall mortality risk than omnivores in the biggest prospective research on vegetarian diets (2). Regarding protein intakes specifically, a prospective cohort analysis indicated that the consumption of plant-based protein instead of animal-based protein was linked to a significant reduction in overall mortality (3).

On the other hand, environmental health also benefits. Because of the non-efficient transformation from plant to animal resources, plant-based foods have generally lower environmental impacts per gram or calorie than animal source foods (4). The high amounts of greenhouse gas (GHG) associated with meat production, as well as the heavy use of water required to raise meat for human consumption, have been found to significantly increase an individual’s carbon footprint (5). Concerning vegetable alternatives, several studies show that they produce less GHG emissions and other environmental effect categories like eutrophication and acidification compared to animal-based products (6, 7).

In line with this, diverse types of plant protein ingredients are becoming more and more available, which makes it necessary to investigate and propose methods to develop foods that fulfill nutritional and environmental criteria (8) to support the much-needed decrease in consumption of animal-based sources.

Nevertheless, the plant-based products on the market often have lower protein content and especially lower protein quality. Studies suggest that healthy adults following an entirely plant-based diet living in Western countries with good food accessibility are generally not at risk of protein or amino acid deficiency (9). Yet, other population groups can be prone to deficiency in those nutrients, especially when they replace one-to-one products of animal origin with those based on plants. For instance, plant-based beverages that should replace milk deliver fewer essential amino acids (10). Especially for long-term consumption, this might be crucial. A study demonstrated that children who consumed three cups of non-cow milk - including plant-based beverages- were significantly smaller at the age of three compared to those who drank the same amount of cow milk per day (11). Another study concluded that the primary consumption of the current generation of plant-based beverages during childhood is associated with specific types of illness. For instance, rice beverages were linked to protein malnutrition and almond-based beverages to metabolic alkalosis (12). Furthermore, our previous study showed that the protein quality of vegan diets might be of concern when only low-quality protein sources are consumed (13).

Protein quality (PQ) does not only involve amino acid composition, but also bioavailability (14). All proteins provide the nine indispensable amino acids (IAA) that the human body needs for metabolic functions, but the distribution of these compounds in plant proteins is less optimal than the ones coming from animals (9). To produce foods or diets with an optimum amino acid profile and improved protein quality, combining different plant protein sources is needed (15). For instance, a well-known case is the mixture of legumes, which are high in the amino acid lysine but low in cysteine and methionine, plus cereals which are high in cysteine and methionine but low in lysine (16, 17). However, this “formula” is not a rule, and individual amino acid profiles need to be accounted for, as well as its digestibility and grade of processing (18, 19).

Linear programming can be a tool to aid the development of food items with the aforementioned criteria. For instance, to find combinations of protein ingredients with a good protein quality, with the least cost and environmental impact. To this date, few solutions using this approach are available to develop new food products. Brixi (20) created an algorithm to formulate “ready to use food for the treatment of acute malnutrition” by minimizing the cost of the 26 raw materials in the dataset while satisfying the imposed nutritional criteria, crop water footprint, and ensuring a PDCAAS > 95, whereas De Carvalho et al. (21) programmed an algorithm to support the formulation of low-cost porridges nutritionally suitable for 1–2-year-old children living in rural Mozambique, including a constraint for protein quantity (but not quality).

To close that gap, the aim of the present project was to develop a digital tool to support the formulation of plant-based foods with a high protein quality by creating combinations of different plant protein ingredients with customizable constraints such as the weight of the mixture, the number, and type of protein ingredients while ensuring a high to excellent protein quality.

## Materials and Methods

**FIGURE 1:**
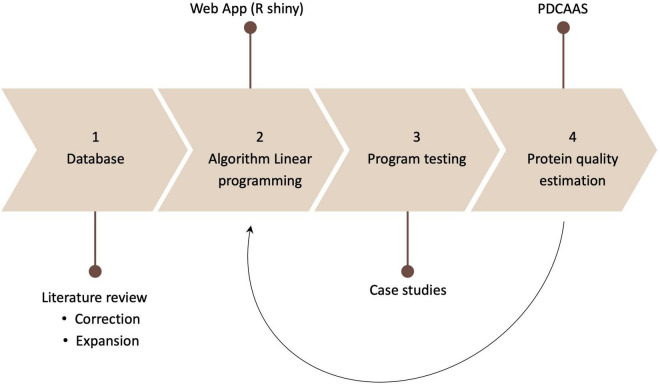
Methodology.

First, a literature review was conducted to expand and revise the existing database containing protein, amino acid contents and digestibility values. Afterward, the algorithm NutriOpt was generated through linear programming in R. The algorithm was then tested using case studies, and the protein quality of the resulting plant protein combinations was estimated. The feedback and suggestions from those tests served to make modifications for the existing application (Figure 1).

### Database Creation

A database of 84 ingredients was set up in an Excel spreadsheet. The ingredients were selected based on their relevance and common use in the meat and dairy analog industry. Each ingredient has a corresponding value on:

• Protein content (g/100 g ingredient).
• Digestible Indispensable Amino Acid (DIAA) content (g/100 g ingredient).

DIAA=ContentofeachIAA(g/100g)×TrueProteinDigestibility(TPD)

• Type of ingredient (i.e., raw grain/seed, protein isolate, protein concentrate).

The values of protein and amino acid content were retrieved in its majority from the USDA database, and the values of TPD from different studies (see Supplementary Material).

Furthermore, it contained the IAA target values (scoring pattern) given by the FAO for adults (14).

### WebApp

The algorithm was written in R (Shiny) using a linear programming approach to generate combinations of ingredients that meet established amino acid targets, protein content, weight, number and type of ingredients, and (optionally) quantity of a mandatory ingredient. The optimization process will start after the selection of one of the objective functions: the optimal solution under the given constraints will be automatically calculated and a report is generated.

The tool has the following two components:

• Excel spreadsheet used as a database.
• R Shiny source code file with the algorithm and the settings for the final user.

The WebApp can be launched directly from the source code in the R console (this solution is only available for the project team) or online as WebApp in a web browser (as a test for guests), which is subjected to Password.

**FIGURE 2:**
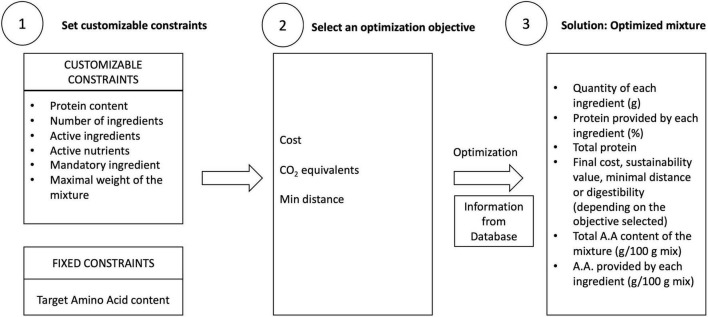
Linear optimization process.

The three main steps of the linear optimization process (Figure 2) of the WebApp based on the algorithm developed with R Shiny are:

Step 1: The optimization process starts by setting a value for each customizable constraint, namely the parameters that the desired blend will have. When a specific number is not set, the algorithm automatically uses the default value (Table 1). There are also fixed constraints, which cannot be changed by the user. They are automatically taken by the algorithm. In this case, the fixed constraints refer to the IAA target values (Table 2).

**TABLE 1:** Customizable constraints of NutriOpt.

	Possible range of selection	Default value
Protein content (g)	0–100	25
Number of ingredients	1–84	5
Activation of ingredients	None – All	All
Activation of nutrients	None – All	All
Mandatory ingredient (“Must be inside product”)	0–4 mandatory ingredients, 0–100% of protein contribution	None
Maximal weight of the mixture (g)	0–300	200

**TABLE 2:** Fixed nutritional constraints according to the IAA scoring pattern for adults [Adapted from FAO (14)].

	His (g)	Ile (g)	Leu (g)	Lys (g)	SAA (g)	AAA (g)	Thr (g)	Trp (g)	Val (g)
For 25 g of protein	0.375	0.75	1.475	1.125	0.55	0.95	0.575	0.15	0.975
For 1 g of protein	0.015	0.03	0.059	0.045	0.022	0.038	0.023	0.006	0.039

Step 2: An optimization objective is selected: minimal distance (optimized allowed residuum), minimum cost, or minimum CO_2_ equivalents. The last two objective functions are fully implemented in the algorithm, but the database currently contains only arbitrary values to test the correct functionality in the App, therefore in the case studied and presented here, we limit ourselves to the test only the objective function minimal distance.

**FIGURE 3:**
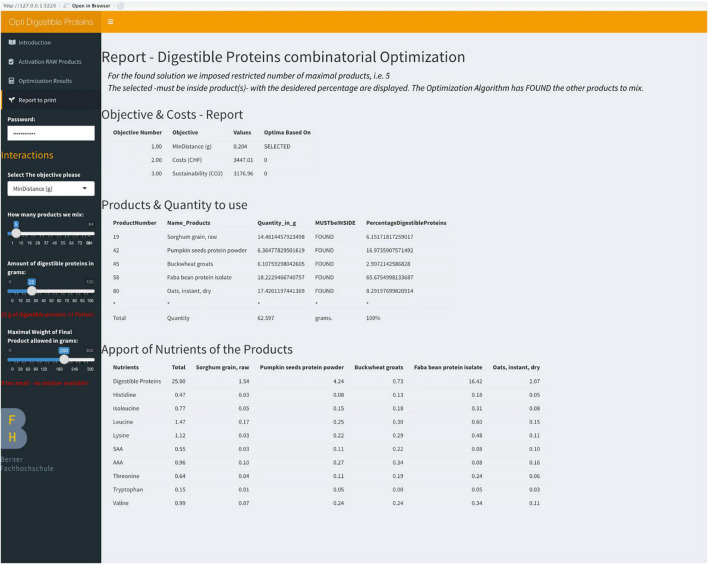
Dashboard of NutriOpt.

Step 3: The algorithm shows the optimal solution (if found) in the dashboard (Figure 3).

### Case Studies

To test NutriOpt, case studies based on plant-based commercial products were used to demonstrate the potential of a real application for the food industry. The main goal was to obtain a combination of ingredients with high to excellent protein quality (PDCAAS > 75) while maintaining similar characteristics (% protein, % protein sources) to market products.

#### Protein Quality Optimization of an Oat Drink

Based on a commercial oat drink that contains 0.3 g protein/100 g product and the following ingredients: Water, oats flour 13%, hazelnut paste 2%, salt. The objective was to reformulate the plant-based drink to obtain a product with 3 g of protein (similarly to the protein contribution of cow’s milk) that still had oats as the main ingredient but substituting the hazelnut paste by two other protein sources derived either from nuts or from seeds. The maximal weight of the mixture was set to 15 g, similarly to the original product. To maintain oat flour as the main ingredient, a constraint of “mandatory ingredient” was set (Table 3).

**TABLE 3:** Constraints to optimize two commercial products.

	Oat drink	Yogurt analog
Protein content	3 g	3 g
Number of ingredients	3	2
Maximal weight of the mixture	15 g	11 g
Active ingredients	Oat flour, nuts, and seeds (flours and press cakes)	Oat flour, pea protein isolate, oat protein concentrate, oat protein isolate, chickpea protein isolate
Mandatory ingredients	Oat flour (45% of protein contribution)	NA

#### Protein Quality Optimization of a Yogurt Analog

Based on a commercial yogurt analog whose label declares 0 g protein and the following ingredients: water, oat flour 11%, coconut fat, waxy maize starch, thickener (E 412). The objective was to reformulate the product to obtain a product with high to excellent PQ and 3 g of protein (similarly to the protein contribution of regular plain yogurt) and ingredients used already for yogurt analogs (the ingredient of several brands of yogurt analog were consulted and from then, the active ingredients selected) and maintaining oat flour in the list of ingredients. The maximal weight of the mixture was set to 11 g, as the original product (Table 3).

### Analysis of Optimized Mixtures

#### Compliance With Constraints

The characteristics of the optimized blend were assessed to verify the compliance with the constraints set. The following checklist was used.

• The ingredient selected “must be inside” is included in the mixture in the indicated percentage.
• The number of ingredients complies with the selected criteria.
• The weight of the mixture complies with the constraint set.
• The indispensable amino acid target is reached (amino acid score).
• The selected amount of protein is reached.
• The activation and inactivation of products is correct as selected by the user.
• The activation and inactivation of nutrients is correct as selected by the user.

#### Protein Quality

The protein quality of the optimized mixtures was estimated with the Protein Digestibility Corrected Amino Acid Score method (PDCAAS) described by FAO (14) using the amino acid content and digestibility of the raw materials:

PDCAAS= mgoflimitingaminoacidin 1goftestproteinmgofthesameaminoacidin 1gofreferenceprotein×Truefecaldigestibility

## Results

It was possible to automatically consider digestibility without having to add an additional constraint, while ensuring a good to excellent protein quality in all the mixtures produced. There was full compliance with the constraints, and no error messages appeared nor were other errors detected. In all assessments the PDCAAS was over 100, indicating an excellent protein quality regardless of the percentage of protein in the mixture.

### Protein Quality Optimization of an Oat Drink

**FIGURE 4:**
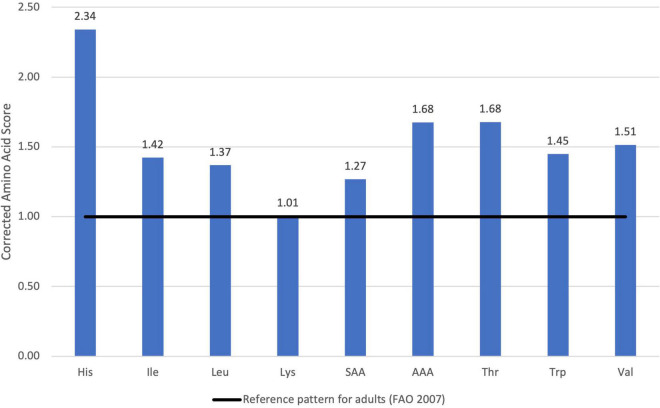
Corrected Amino Acid Score of optimized formula for oat drink.

The optimized mixture complied with all the constraints and the result was a combination of oat flour, hemp press cake and pumpkin seed flour (Table 4). This combination has a similar amount of oat flour compared to the original product. Regarding protein quality, the PDCAAS was 100 (101 not truncated) (Figure 4). Thus, it is classified as high quality. Lysine had the lowest value of the corrected amino acid score, while histidine had the maximal (2.34 times more than the reference pattern).

**TABLE 4:** Optimized mixture for oat drink.

	Optimized formula	
Ingredient	Quantity (g)	Protein contribution (%)
Oat flour	10.2 (70%)	45.0%
Hemp press cake	2.6 (18%)	47.1%
Pumpkin seed flour	1.6 (12%)	7.9%
*Total*	*14.4 (100%)*	*100.0%*

Compared to the reference product, the optimized formula has a similar quantity of protein ingredients, whereas the protein content was increased from 0.3 to 3 g, respectively. On the other hand, the protein quality also increased: from 60 to 101 (non-truncated value) and from 57 to 94 for adults (>18 years) and older children and adolescents (4–18 years), respectively (Table 5).

**TABLE 5:** Comparison of optimized formula versus original product.

	Original	Optimization
Protein content	0.3 g	3 g
Weight of the mixture	15 g	14.4 g
List of ingredients	Water, oat flour (13%), hazelnut paste (2%), salt	Water, oat flour (10%), hemp protein (2.6%), pumpkin seed protein (1.6%), salt
PDCAAS	60* (>18 years) 57* (4–18 years)	101 (not truncated) (>18 years) 94 (4–18 years)

*Own calculation based on the protein digestibility of raw walnuts and amino acids values from USDA database.

### Protein Quality Optimization of a Plant-Based Yogurt Analog

**FIGURE 5:**
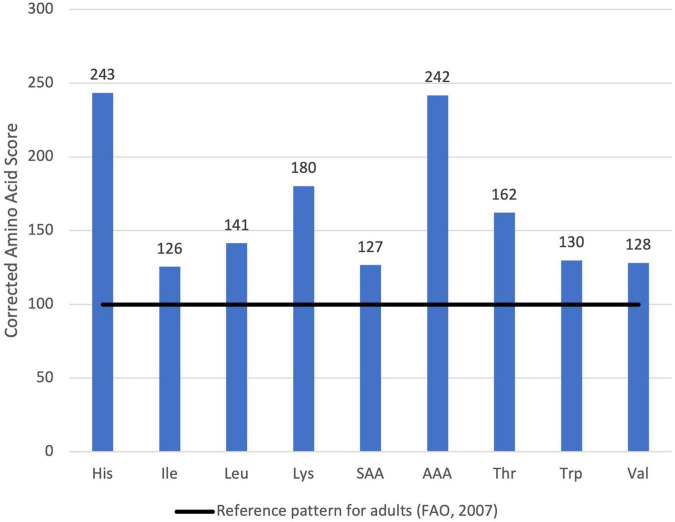
Corrected Amino Acid Score of optimized formula for yogurt analogue.

For this case study, NutriOpt generated a combination of oat flour and pea protein isolate (Table 6), which is in line with the constraints set (only 2 ingredients, 11 g of total weight) and it was not necessary to set oat flour as a mandatory ingredient since this was automatically selected for the optimal mixture. Regarding protein quality, the lowest protein corrected amino acid score was 126 for Isoleucine (Figure 5). Therefore, as values in PDCAAS are truncated, the final protein quality is reported as 100. All IAA exceed the target set for adults. For the category of older children and adolescents (4–18 years) the estimated PDCAAS is 120, while for preschool children (1–2 years) the PDCAAS is 105 (shown in Supplementary Material).

**TABLE 6:** Optimized mixture for yogurt analog.

	Optimized formula	
Ingredient	Quantity	Protein contribution (%)
Oat flour	8.62 g (78%)	62%
Pea protein isolate	2.38 g (22%)	38%
*Total*	*11 g (100%)*	*100%*

The optimized formula provides 3 g of protein versus the original that declares 0 g. The main protein ingredient in terms of quantity is still oat flour, and by adding 2.4% of pea protein isolate to the formulation, it was possible to increase the protein quality (Table 7).

**TABLE 7:** Comparison of optimized yogurt analog formula versus original product.

	Original	Optimization
Protein content	0 g	3 g
Weight of the mixture	11 g	11 g
List of ingredients	Water, oat flour 11%, coconut fat, corn starch, thickener (E 412)	Water, oat flour 8.6%, pea protein 2.4%, coconut fat, corn starch, thickener (E 412)
PDCAAS	0	126 (not truncated) for >18 years 120 (not truncated) for (4–18 years)

## Discussion

The NutriOpt program can generate blends with a protein quality that is comparable to that of milk protein concentrate (PDCAAS = 125 for the 0.5–3-year-old child) (22). Conversely, the PDCAAS of the raw materials used for some commercial plant-based beverages has been reported to be significantly lower than their animal-based homologs. Namely 68 for quinoa, 63–66 for hemp, 45–60 for oat, 54 for rice, and 30 for almond (23).

In the case studies, the optimized formulas had similar characteristics to the market products regarding proportion and type of protein ingredients, which can facilitate the development process of optimized products. For the yogurt analogs, pea was selected as the complementary protein source. This is a classic example of amino acid complementarity, where a legume, which has a high amount of lysine, but low content of SAA is combined with a cereal that possesses a considerable content of SAA and a low lysine content, resulting in a blend with improved protein quality (16, 17). Nevertheless, the proportion of the protein sources should not be disregarded because it is not a rule that a legume plus a cereal will have higher PQ scores (24, 25). In both case studies, some amino acids had more than double the amount of the target, but this might be restricted with an additional constraint. Yet, it is important to mention that the addition of a new constraint may decrease the possibility to find a solution. Besides, the constraints need to be logically set. For instance, for obvious reasons, the amount of total protein must be lower than the maximal weight of the mixture. Otherwise, there are no possible solutions.

Limitations inherent to the PDCAAS methodology also limit the interpretation of the results in this study. This approach aims to forecast the utilization of dietary protein to estimate to which degree it can meet the demand for the amino acids necessary for maintenance functions (26). However, it is subject to several limitations. For instance, the erroneous assumption that the digestibility of the crude protein is equal to that of each individual IAA. The absorption rate of these compounds can largely vary between each other, especially in the presence of antinutritional factors (27). Another important shortcoming is the measurement of digestibility along the whole digestive tract (i.e., fecal) which is considered to overestimate the absorption (28). Although the procedure suggests that digestibility values derive from *in vivo* rat analysis, some of the data here were extracted from *in vitro* studies due to availability reasons. Yet, studies indicate that *in vitro* assays can provide an accurate estimate of the True Protein Digestibility (29–31). Despite the disadvantages, PDCAAS has been valuable in practice (26) and has been chosen in this work due to its practicality, acceptability, and the wider availability of data than its counterpart DIAAS.

Having acknowledged the latter drawbacks, it is important to remark that even though the algorithm is capable to suggest mixtures that reach the digestible amino acid targets and thus a theoretical high PDCAAS, the quality of the results will depend on the quality of the data. Should the database have an erroneous, arbitrary, or missing value, the accuracy of the outcome might be compromised.

Another limitation was the availability of data. To this date, not all the digestibility values and threonine content of the ingredients in the database have been found in the literature: some of them are arbitrarily set or based on assumptions. Therefore, if NutriOpt is to be used in a real setting, these items should be deactivated. It is recommended to update the database information according to the specific ingredient data of the final user.

Moreover, the calculated quality of the protein blends is based on the ingredients as raw materials. However, further processing, the structural organization of the food matrix, and interactions between other ingredients can greatly influence the amino acid content and digestibility of the final product (8, 19, 32, 33). Finally, the sensory and technological performance of the protein combinations provided by NutriOpt is not addressed in the present study and should be subject to test in future research.

A strength of the present project is, however, that the level of refinement in different protein sources was considered. For instance, the availability of one source in different presentations such as raw flour, defatted flour, concentrate, isolate, and cooked form. This individualization provides a more accurate panorama of the amino acid content and bioavailability of the ingredients.

Another advantage is that the database contains a broad range of ingredients that are already being used in the food industry, as well as novel protein sources such as hemp press cake and microalgae protein. This allowed recreating optimized products that are already commercially available. Additionally, most of the data were retrieved from ingredients intended for human food consumption, while other studies in PQ optimization such as that developed by Herreman et al. (24) use datasets from animal nutrition studies, mostly in their raw form.

Given that the algorithm gives a constraint already in the digestible IAA, values not only of good but of excellent protein quality (PDCAAS > 100) are consistently obtained (The values were not truncated for purposes of analysis).

In the future, a wider number of ingredients, organized in categories and groups, can be added. In the view of the circular economy, by-products of oil extraction such as press cakes are a good alternative source of protein. Other optimization objectives can be used such as minimizing cost or CO_2_ equivalent, provided that real values are incorporated in the database. Due to time constraints, this work does not include them, but future research can consider them. The estimation of protein quality with the DIAAS method can be implemented when the values of ileal amino acid digestibility are available from new studies.

A version with whole plant-based food (cooked legumes, nuts, cereal-based products) can be set up, prospectively supporting a transition to healthy and sustainable diets, or directed to populations with risk from protein deficiency.

This work is focused on protein, but additional nutritional parameters might be added such as energy, carbohydrates, fat, and fiber, having in consideration that the more constraints present, the smaller number of available solutions.

Assessment of the functionality of the ingredients. It is not clear whether the mixtures are technologically or sensorially appropriate.

Finally, it might be possible to do an automated customization of diets based on specific needs: specific setups for different types of categories of people can be implemented by optimizing the intake of proteins taking into account that the final result is for an athlete, an elderly person, or a person with allergies or food intolerances.

## Conclusion

There is an increasing number of plant protein ingredients to enable the transition toward more sustainable food production, and such resources must be utilized in a way that provides the consumer with nutritious food alternatives. A constant matter of concern in the transition toward a more plant-based diet is the question of whether the requirements of essential amino acids are being met. A digital tool that provides a combination of ingredients with good and excellent protein quality was developed. This can facilitate the formulation of nutritious products such as, but not limited to dairy/meat analogs, and snacks with a good to excellent protein quality, especially for populations at risk of amino acid deficiency. The novelty of this project is the inclusion of raw materials suitable for the food industry for meat and dairy analogs (protein concentrates, isolates, flours), and the relatively large choice of ingredients in the database: from common ingredients such as soy products to more innovative ones such as microalgae or press cakes. Moreover, the changes in digestibility and amino acid composition are considered due to the inclusion of foodstuffs with different grades of processing, and the digestibility values are taken -when available- from human studies. This program has a great potential for expansion both for food development and for diets. For instance, by amplifying the number and type of ingredients, or by adding data on environmental impact or raw material cost. The question of technological performance remains since the combinations have not been proven in practice, but the customization functions and expert technical insight can serve to deactivate or limit ingredients that, for example, have undesired off-flavors or that provide undesired texture. The algorithm can potentially create solutions with minimum cost or environmental impact, provided adequate data is fed into the database. It is necessary to improve the protein quality of plant-based alternatives of the second generation as long as the consumer uses them really as replacements of the products that originate from animals, especially on long-term. Otherwise, the whole diet must be adapted. Our model can be also used to optimize mixed meals and whole diets in the future.

## Data Availability Statement

The original contributions presented in the study are included in the article/Supplementary Material, further inquiries can be directed to the corresponding author.

## Author Contributions

ZR prepared the first version of the manuscript in the frame of her Master thesis, tested the program, and updated the database. KK-B had the idea for the project, conducted the first tests, did the supervision of the nutritional part of the project, and corrected the manuscript. RR was responsible for the digital optimization and the supervision of this part. All authors contributed to the article and approved the submitted version.

## Conflict of Interest

The authors declare that the research was conducted in the absence of any commercial or financial relationships that could be construed as a potential conflict of interest.

## Publisher’s Note

All claims expressed in this article are solely those of the authors and do not necessarily represent those of their affiliated organizations, or those of the publisher, the editors and the reviewers. Any product that may be evaluated in this article, or claim that may be made by its manufacturer, is not guaranteed or endorsed by the publisher.

## Supplementary Material

Supplementary Table 1 Nutritional information of the protein sources and references.
